# Perceived Labor Control Among Latinas: Examining the Role of Pregnancy Loss, Prenatal Depression, and Childbirth Pain Severity

**DOI:** 10.1111/jmwh.70145

**Published:** 2026-06-23

**Authors:** Sandraluz Lara‐Cinisomo, Biniyam Melesse

**Affiliations:** ^1^ Department of Health and Kinesiology University of Illinois at Urbana‐Champaign Champaign Illinois; ^2^ Division of Maternal Child & Family Health Illinois Department of Public Health Springfield Illinois

**Keywords:** childbirth, depressive symptoms, labor pain, latinas, perceived control, pregnancy loss

## Abstract

**Introduction:**

Prenatal mental health, perception of pain, and pregnancy histories can affect a birthing person's sense of control during labor. Although recent research has focused on cesarean births, less attention has been given to the role those factors play in perceived control during vaginal births. These gaps are particularly critical for Latinas, who experience health care intrapartum disparities in the United States. This study addresses these gaps by examining how prenatal depression symptoms, history of pregnancy loss, and pain severity are related to perceived control during labor among Latinas birthing vaginally. The findings may inform intrapartum care for a patient population at increased risk of health care inequities.

**Methods:**

This secondary data analysis included 119 self‐identified Latina women who had a vaginal childbirth in North Carolina. Hierarchical linear regressions tested associations between prenatal depressive and anxiety symptoms, history of pregnancy loss, labor pain severity, and perceived control of labor.

**Results:**

A history of pregnancy loss was associated with lower perceived control during labor across all models, even after controlling for covariates, mental health, and perinatal variables (*B =* −0.450; *P* =  .024). Prenatal depressive symptoms were also a robust predictor, showing an inverse association with perceived control of labor in the final model with all predictors (*B* = −0.076; *P* =  .014). Lastly, there was a significant and negative association between pain severity and perceived control of labor, even after controlling for covariates, histories of pregnancy loss, prenatal depressive symptoms, and analgesia use in labor (*B =* −0.355; *P* <  .001).

**Discussion:**

Latinas in the United States encounter health care inequalities during and after childbirth. The findings underscore the need for clinicians to screen for depressive symptoms, discuss patients’ pregnancy histories, support pain management during labor, and implement shared decision‐making protocols.

## INTRODUCTION

A birthing person's perceived control over labor during childbirth is a crucial predictor of overall satisfaction with the childbirth experience and subsequent health outcomes.^1^ Perceived control of labor includes the person's sense that they can manage, respond to, and participate in the labor process rather than control the largely involuntary processes of labor. The individual's perceived control is important to the individual's well‐being because, as provided in stress and coping theory, it affects how the birthing person appraises childbirth labor as well as their perceived ability to respond to its demands; low levels of perceived control increase the risk of adverse mental health outcomes, including depression and anxiety.^2^, ^3^ Perceived control of labor is particularly important during vaginal childbirth because it offers an opportunity to explore the childbirth experience. This is because birthing people may be more active participants in vaginal births because it involves different forms of physical responses and decision‐making compared with cesarean births. However, one's perceived control of labor is not solely dependent on one's appraisal of the stressor or situation, as the theory suggests. Instead, proximal factors may affect one's perceived control during vaginal childbirth, which we explore in this study.

1 **QUICK POINTS**

✦ A history of pregnancy loss and prenatal depression symptoms were inversely associated with perceived labor control among the Latinas in this study.
✦ Greater labor pain severity was negatively associated with perceived control of labor in our Latina sample.
✦ The findings underscore the importance of screening for prenatal depression, discussing pregnancy histories, and supporting effective labor pain management among Latinas.
✦ The study addresses a significant gap by providing empirical data to enhance personalized perinatal care for Latinas.

Examining vaginal birth experiences among Latinas is critical, given structural inequalities in health care and birthing. A Latina is a woman who identifies with or is descended from Latin American cultural, national, or ancestral backgrounds.^4^ The term *Latina* will be used in this study unless specific terminology from the referenced literature (eg, *Hispanic*) is necessary. Inequities in perceived labor control have been documented; one study of 5,750 patients showed that Latina women reported significantly lower perceived labor control compared with White, non‐Latina women.^5^ Furthermore, Latinas who are pregnant and postpartum may be at greater risk for gendered racism compared with White, non‐Latina women,^6^ and many Latina women report perceived discrimination during intrapartum.^7^ Despite the importance of understanding immediate and influential factors, few studies have explored how these determinants affect Latinas’ perceived control during vaginal births. This knowledge is essential for informing clinical care and future research. Our study focuses on pregnancy loss, psychological factors, and perinatal factors in a sample of Latinas to address gaps in the literature that can help clinicians and researchers provide more targeted and responsive support to enhance Latinas’ sense of control during labor.

There is limited information about the link between a history of pregnancy loss and perceived control during labor. This area of research is crucial because of the potential for increased stress and anxiety during childbirth; patients who have faced a pregnancy loss may feel a heightened need to control the pregnancy and birth of their child.^8^, ^9^ However, some studies have found no association between histories of pregnancy loss and perceived control of labor.^5^ Latinas with histories of pregnancy loss may suffer in silence or isolation and experience other risk factors,^10^ yet they are underrepresented in research on pregnancy loss. Examining the association between pregnancy loss and perceived control of labor among Latinas is essential to offer intrapartum support that recognizes critical health histories, intersecting vulnerabilities, and positive birth experiences.

Perceived control of childbirth labor is also an important function of prenatal mental health, such as depression and anxiety. Prenatal depression and anxiety can adversely affect the birthing person and their child, including preterm birth, severe maternal morbidities, and mortality.^11^ There is evidence that prenatal depression and anxiety symptoms are associated with lower perceived control during labor.^12^, ^13^ However, studies that have included racially diverse samples of women have not examined whether associations differ by race or ethnicity.^13^ This gap is crucial because Latinas in the United States often face structural factors, such as implicit bias, that can hinder their sense of control. Cultural norms, such as Marianismo, which emphasizes self‐sacrifice and deference, may further diminish their sense of control during childbirth.^14^ These issues can be complicated by symptoms of depression or anxiety, which may already lead to reduced self‐efficacy.^15^

Pain during childbirth can also affect perceived control during labor. Research on perceived control during labor indicates that childbirth pain severity is a significant and predictive factor of childbirth experiences, including perceived control over labor.^5^ Mallett et al found that women who experienced moderate to high pain (≥8) experienced less perceived control of labor than those who reported lower levels of pain.^5^ Latinas may face additional challenges during childbirth due to structural and interpersonal barriers, including language barriers, cultural expectations, beliefs about pain, preferences for pain management, experiences of discrimination, and health care disparities.^7^, ^16^, ^17^

To address gaps in the existing literature, we conducted a secondary data analysis involving 119 Latinas who had vaginal births.^18^ We examined whether prenatal depressive and anxiety symptoms, pain severity, and a history of pregnancy loss were significantly and inversely associated with perceived control during labor. The goal is to inform clinicians in providing personalized and responsive intrapartum support to Latinas facing complex structural and psychosocial challenges.

## METHODS

### Sample

Three hundred and twenty‐five individuals between 31 and 33 weeks’ gestation were approached by trained research staff for the parent study at a local hospital, and 289 were enrolled.^18^ Of those, 153 self‐identified as Hispanic or Latina in response to the following question: “Are you Hispanic or Latina?” Respondents selected “Yes” or “No” (hereafter, Latinas). Eleven participants were excluded because of incomplete data necessary for the current study. Of the 142 with complete prenatal and 6‐week postpartum data collected during home visits, 23 had a cesarean birth and were excluded. The final sample included 119 women who had a vaginal birth.

The data were collected between July 2008 and August 2012 as part of a more extensive study conducted at the University of North Carolina at Chapel Hill on thyroid function in women.^18^ These data are still relevant today because Latinas in the United States continue to face health care disparities and unequal childbirth experiences.^19^

### Sampling Procedures

Women were included in the original study if they were aged between 18 and 45 years. Women were excluded if they had a lifetime history of psychotic, bipolar, cyclothymic, somatoform, or dissociative disorder according to the *Diagnostic and Statistical Manual of Mental Disorders, Fourth Edition* ^20^; a body mass index greater than 35 or less than18; experienced chronic or acute serious clinical illness or pregnancy complication; a substance dependence or eating disorder in the last 2 years; or used street drugs more than 3 days per week or consumed alcoholic beverages for more than 2 drinks per day during the current pregnancy. Also, women who could not speak or write English or Spanish were excluded. Women were also excluded if they had chronic or severe health or pregnancy complications, such as heart disease, cancer, diabetes, or recent surgery, and if they smoked over 10 cigarettes per day.

### Human Ethics and Consent to Participate

Written informed consent was obtained from all participants in the original study. Institutional approval for human research was obtained from the University of North Carolina at Chapel Hill, where the original study was conducted. The institutional review board waived ethical review and approval for this study and did not assign a protocol code to the approval document for the parent study. The study was conducted in accordance with the 1964 Declaration of Helsinki and subsequent amendments. This secondary data analysis did not require ethical approval from the University of Illinois Urbana‐Champaign because all the data provided by the University of North Carolina at Chapel Hill were deidentified. The Strengthening the Reporting of Observational studies in Epidemiology (Supporting Information: Appendix S1) guideline was followed in generating this report.^21^

### Measures

All the data were self‐reported and collected by trained research staff during home visits. Demographic data and prenatal depressive and anxiety symptoms were collected at 35 to 36 weeks’ gestation in the parent study using the measures below. Labor‐related factors (ie, labor analgesia, labor pain intensity, and perceived labor control) were collected from participants at 6 weeks postpartum in the original study. All the measures were available in Spanish or English.

#### Demographic Data

Using a self‐administered instrument, demographic data were collected from pregnant women, including age, marital status, education level, household income, and employment status. Women also reported the number of previous pregnancies, live births, stillbirths, miscarriages, and abortions. Pregnancy loss was captured by combining histories of stillbirth (ie, loss of a fetus after 20 weeks’ gestation) or miscarriage (ie, loss of a fetus before 20 weeks’ gestation). Women also reported whether they took birthing classes during the current or a previous pregnancy.

#### Depressive Symptoms

To determine the presence of prenatal depressive symptoms, participants were asked to complete the Edinburgh Postnatal Depression Scale (EPDS), a valid screener used during the prenatal and postpartum period.^22^, ^23^ Scores range from 0 to 30, with higher scores indicating more symptomatology. The risk for depression was determined using a cutoff score of 10 or greater, which is recommended for major and minor depression and has been used in previous studies of Latinas.^24^, ^25^ The internal consistency of the EPDS is high (α =  .83).^26^ The EPDS scores were modeled as a continuous variable, and cutoff scores were used for descriptive purposes.

#### Anxiety Symptoms

Participants also completed the State‐Trait Anxiety Inventory (STAI), a 40‐item scale comprising 2 self‐report subscales assessing current and general anxiety.^27^ The STAI trait subscale (STAI‐T) was administered to determine prenatal anxiety symptoms in the past week. Scores range from 20 to 80,^27^, ^28^ with higher scores indicating more anxiety symptoms. The STAI‐T scores were modeled as a continuous variable. For descriptive purposes, the STAI‐T score was classified into high‐anxiety and low‐anxiety groups according to the recommended cutoff score of 40.^29^ The STAI‐T has an acceptable internal consistency (α =  .72).^30^ The STAI‐T has been used to examine anxiety in perinatal English‐speaking and Spanish‐speaking women.^25^

#### Labor Analgesia

The type of labor analgesia was self‐reported at 6 weeks postpartum. Participants responded to the following question: “For pain relief during labor, did you receive any of the following: Injection or Intravenous (IV) pain medication, Epidural or Spinal (shot given in your back), or any other medication for pain, please specify?” Women who reported receiving an IV and epidural or spinal analgesia were recoded as receiving “both” analgesics. We also created a variable to capture whether any (IV or spinal blocker) labor analgesia was used. Because of the small sample size, other medications reported by participants (eg, ibuprofen) were not included in the analysis.

#### Labor Pain Severity

Recalled labor pain was self‐reported using a single item: “Which of the following words best describes the pain you experienced during childbirth?” Participants responded on a 5‐point Likert‐type scale ranging from 1 (no pain) to 5 (extremely severe pain). Verbal rating scales are reliable single‐item measures of individuals’ pain.^31^, ^32^, ^33^ Although an 11‐point pain scale (ranging from 0 to 10) is commonly used, some studies have shown that shorter scales, such as a 5‐point scale, can also be valid.^34^, ^35^ These shorter scales effectively capture patients' experiences and correlate with clinical outcomes, including changes in pain and disability.^34^, ^35^

#### Perceived Control of Labor

Participants reported perceived control of labor by indicating the extent to which they felt “in” and “out” of control during labor using a scale from 1 (“completely out of control”) to 4 (“completely in control”). There is evidence of the validity of a single control item.^36^

### Statistical Analysis

The sample was characterized using frequencies for categorical variables and means and SDs for continuous variables. There were no missing data. Bivariate correlations were performed to test the associations between prenatal depressive and anxiety symptoms, pregnancy loss, and perinatal variables. Hierarchical linear regressions were conducted to identify the variables associated with perceived control of labor. Covariates (ie, age, marital status, income, education, employment status, parity, and attended birthing classes) were entered in block 1. In block 2, pregnancy loss was added with the block 1 covariates. Pregnancy loss was added, given its link to perceived control of labor, to determine whether it explained additional variance in the model. Depression and anxiety symptom scores were included in block 3 to assess their association with perceived control of labor and determine whether they explained additional variance in the model beyond block 2. Block 4 included pain severity, given the cited literature. Using analgesics or other pain relief would increase perceived control during labor. Pain relief has been found to contribute to perceived control of labor.^37^ We used “any analgesic” in the models because the results did not differ between individual or combined analgesics. Bootstrapping was not used because the results did not differ in the parametric models. Significance was determined at the  .05 level, and 95% CIs were estimated. Analyses were conducted in IBM SPSS 29.0.

### Positionality and Reflexivity Statement

The secondary data analysis was conducted by the 2 authors, both of whom have research experience with women who are perinatal. Preliminary findings were disseminated and discussed at a professional conference attended by perinatal mental health scholars and other members of the lead authors’ research laboratory. Reflexivity was maintained through ongoing evaluations of the researcher's positionalities and the perinatal pain literature. The authors are trained perinatal mental health researchers; one identifies as Latina and the other as an African American man.

## RESULTS

Table 1 presents the descriptive statistics for the sample. Nearly 22% of the participants met the EPDS cutoff score greater than 10, indicating the presence of depression symptoms. Additionally, one‐third of the sample experienced high anxiety, as determined by the STAI‐T score exceeding 40. The mean (SD) pain severity was 4.26 (0.96), categorizing it as “severe.” Furthermore, the mean (SD) perceived control during labor was 2.86 (0.99), suggesting participants felt “somewhat in control.”

**Table 1:** Descriptive Statistics of the Sample (N = 119)

Characteristic	Value
**Age, mean (SD) [range], y**	27.08 (5.36) [18‐40]
**Marital status, n (%)**	
Married/cohabitating	98 (82.4)
Single/intimate relationship	21 (17.6)
**Education, n (%)**	
Less than high school	29 (24.4)
High school or more	90 (75.6)
**Income, n (%)**	
≤$20,000	62 (52.1)
>$20,000	57 (47.9)
**Not employed at enrollment, n (%)**	60 (50.4)
**Primiparous, n (%)**	18 (15.1)
**Attended birthing classes, n (%)**	83 (69.7)
**History of pregnancy loss, n (%)** ^a^	31 (30.7)
**Depression symptoms**	
Mean prenatal score, mean (SD) [range]	6.28 (4.50) [0‐21]
Met the cutoff score during pregnancy, n (%)	26 (21.8)
**Anxiety symptoms**	
Mean prenatal score, mean (SD) [range]	35.18 (9.34) [20‐69]
Met the cutoff score during pregnancy, n (%)	37 (31.1)
**Labor analgesia, n (%)**	
Any labor analgesia	77 (64.7)
*Intravenous pain blocker* ^b^	47 (61.0)
*Epidural/spinal analgesia pain blocker* ^b^	14 (18.2)
*Intravenous and Epidural* ^b^	16 (20.7)
No labor analgesia	42 (35.3)
**Labor pain severity, mean (SD) [range]**	4.26 (0.96) [1‐5]
**Perceived control of labor, mean (SD) [range]**	2.86 (0.99) [1‐4]

a Among women who were multiparous.

b Among those who reported any labor analgesia.

Bivariate associations between prenatal symptoms, labor‐related variables, and perceived control during childbirth are presented in Table 2. There was a significant and positive correlation between prenatal depressive symptoms and prenatal anxiety symptoms (*r*_119_,  .792; *P* <  .001). There was a significant and negative correlation between prenatal depressive and anxiety symptoms and perceived control of labor (*r*_119_ of −.295 [*P* =  .001] and *r*_119_ of −.219 [*P* =  .017], respectively). Additionally, labor pain severity was significantly and negatively associated with perceived control of labor (r_119_, −.345; P <  .001). The point‐biserial correlation further indicated that the use of any labor analgesia was negatively associated with perceived control of labor (*r*_119_, −.215; *P* =  .019).

**Table 2:** Correlation Coefficients for Prenatal Symptoms, Labor‐Related Variables, and Perceived Control of Childbirth Labor

Variable	1	2	3	4	5
Prenatal depressive symptoms (1)	1				
Prenatal anxiety symptoms (2)	.792^a^	1			
Use of any labor analgesia (3)	.101	.119	1		
Labor pain severity (4)	.073	.074	.054	1	
Perceived labor control (5)	−.295^b^	−.219^c^	−.215^c^	−.345^a^	1

a *P* <  .001.

b *P* <  .01.

c *P* <  .05.

### Associations Between Prenatal Symptoms, Childbirth Labor‐Related Variables, and Perceived Control of Childbirth Labor

The results from the hierarchical linear regression analyses reveal that none of the covariates in block 1 were significantly associated with perceived control of labor (see Table 3). Including a history of pregnancy loss in block 2 explained 6.4% of the model's variance and was significantly and negatively associated with perceived control of childbirth labor (*B* = −0.451; SE, 0.215; *P* =  .038; 95% CI, −.876 to −.025).

**Table 3:** Hierarchical Regression Results for Perceived Control of Childbirth Labor

Characteristic	*B*	SE *B*	β	T	95% CI	R^2^	R^2^_Adjusted_	ΔR^2^_Adjusted_
**Block 1**						.027	−.035	.027
Age	−.005	.018	−.025	−.249	−.041 to .032			
Single	−.145	.338	−.043	−.430	−.814 to .524			
Less than high school	−.063	.221	−.027	−.283	−.501 to .376			
$20,000 income or less	−.121	.194	−.062	−.623	−.507 to .264			
Unemployed during pregnancy	−.187	.189	−.095	−.989	−.562 to .188			
Primiparous	.150	.270	.055	.555	−.385 to .685			
Attended birthing classes	.160	.205	.075	.781	−.246 to .567			
**Block 2**						.064	−.004	.038
Age	−.004	.018	−.022	−.230	−.040 to .031			
Single	−.201	.334	−.059	−.601	−.862 to .461			
Less than high school	−.061	.218	−.027	−.280	−.493 to .371			
$20,000 income or less	−.109	.192	−.056	−.570	−.489 to .270			
Unemployed during pregnancy	−.195	.187	−.099	−1.044	−.564 to .175			
Primiparous	.006	.275	−.002	.022	−.539 to .551			
Attended birthing classes	.122	.203	.057	.601	−.280 to .524			
Pregnancy loss	−.451^c^	.215	−.202	−2.100	−.876 to −.025			
**Block 3**						.157	.079	.093
Age	.001	.017	.006	.062	−.033 to .035			
Single	.052	.328	.015	.160	−.597 to .702			
Less than high school	−.049	.209	−.021	−.233	−.464 to .366			
$20,000 income or less	−.110	.184	−.056	−.599	−.475 to .254			
Unemployed during pregnancy	−.227	.180	−.116	−1.265	−.583 to .129			
Primiparous	−.058	.265	−.021	−.218	−.583 to .468			
Attended birthing classes	.171	.195	.080	.879	−.215 to .558			
Pregnancy loss	−.466^c^	.208	−.208	−2.242	−.878 to −.054			
Prenatal depressive symptoms	−.074^c^	.033	−.338	−2.257	−.139 to −.009			
Prenatal anxiety symptoms	.003	.016	.024	.164	−.028 to .034			
**Block 4**						.282	.201	.125
Age	.010	.016	.055	.610	−.023 to .043			
Single	.171	.313	.050	.545	−.450 to .791			
Less than high school	−.096	.196	−.042	−.490	−.484 to .292			
$20,000 income or less	−.140	.173	−.071	−.813	−.483 to .202			
Unemployed during pregnancy	−.169	.168	−.086	−1.005	−.503 to .165			
Primiparous	−.152	.249	−.055	−.610	−.645 to .341			
Attended birthing classes	.128	.184	.060	.697	−.236 to .492			
Pregnancy loss	−.450^c^	.197	−.201	−2.286	−.841 to −.060			
Prenatal depressive symptoms	−.076^c^	.031	−.348	−2.494	−.137 to −.016			
Prenatal anxiety symptoms	.007	.015	.068	.485	−.022 to .036			
Any labor analgesia	−.210	.183	−.102	−1.145	−.572 to .153			
Pain severity	−.355^a^	.088	−.346	−4.025	−.530 to −.180			

Any analgesia = any analgesia used during childbirth labor. Pain severity = recalled pain severity during childbirth labor.

a *P* <  .001.

b *P* <  .01.

c *P* <  .05.

In block 3, the addition of prenatal depression and anxiety symptoms further increased the explained variance to 15.7%. Notably, a history of pregnancy loss retained its significance even after accounting for depressive and anxiety symptoms (*B =* −0.466; SE, 0.208; *P* =  .027; 95% CI, −.878 to −.054). Prenatal depressive symptoms were also significantly and inversely associated with perceived control of childbirth labor (*B* = −0.074; SE,  .033; *P* =  .026; 95% CI, −.139 to −.009).

Finally, adding analgesia and pain severity to block 4 increased the explained variance of perceived control of labor from the previous model to 28.2% (see Table 3). Both a history of pregnancy loss and prenatal depressive symptoms remained significant in the model, with each showing a significant inverse association with perceived control of labor. Similarly, there was a significant and negative association between pain severity and perceived control of labor (*B* = −0.355; SE,  .088; *P* <  .001; 95% CI, −.530 to −.180)

## DISCUSSION

Prenatal mental health, pain perception, and perinatal history can influence a woman's sense of control during labor. Most recent research has primarily focused on cesarean births, with less attention given to vaginal births. This gap is particularly significant for Latinas, who experience health care disparities during labor in the United States, including racial and gendered microaggressions, feeling ignored, and delayed pain management.^19^, ^38^, ^39^ We examined whether a history of pregnancy loss, prenatal depressive and anxiety symptoms, and pain severity were associated with perceived control during labor among Latinas with vaginal births to help inform intrapartum care.

The results revealed that a history of pregnancy loss was a consistent and robust predictor of lower perceived control of labor. Across all the hierarchical models, women with a history of pregnancy loss reported significantly lower levels of perceived control of labor than those without such a history. These findings address a critical gap in the literature by incorporating histories of pregnancy loss that are insufficiently examined, particularly among Latinas in the United States, whose reproductive and health experiences are underrepresented in intrapartum research. As noted earlier, Latinas may experience pregnancy loss in silence because of stigma or expectations.^10^ By shedding light on the lingering effects of pregnancy loss among Latinas, our findings bring attention to critical clinical considerations for this population. The findings also help reconcile mixed findings. For instance, Mallette and colleagues did not find a significant association between pregnancy loss and a sense of childbirth control in a racially and ethnically diverse sample that included cesarean and vaginal births 6 to 96 hours after childbirth.^5^ However, they did find that Hispanic/Latina women experienced a lower sense of control during childbirth, which aligns with our findings.^5^

The findings also revealed that higher prenatal depression symptoms were consistently associated with lower perceived control of labor across all hierarchical models while controlling for demographic variables, anxiety, and labor‐related factors. These findings support previous studies showing that depression symptoms hinder women's sense of control during labor.^13^ Although perceived control of labor has not been widely examined in the context of prenatal depression symptoms, existing literature shows that depression compromises self‐agency.^40^, ^41^ Our findings extend this research to the intrapartum context, underscoring the importance of assessing depression symptoms to support women's sense of control during labor. This is particularly critical for Latinas who have an elevated risk of prenatal depression because of complex psychosocial (eg, discrimination) and structural factors (eg, poverty).^42^

Higher pain severity was associated with lower perceived labor control, even after controlling for analgesics. The findings support previous research indicating that women who reported feeling in control of their labor were likelier to report lower pain severity.^43^ Pain is a precursor to labor, and severe pain may impede a sense of control even when pain medication is administered during labor. Pain is complex and can be influenced by other factors, including beliefs about pain, patient preferences, hospital guidelines that determine when and how pain medications are administered, patients’ prior experiences, and the timing of the labor pain measure. How pain is assessed and how labor pain coping options (including pain medications) are offered are critical to understand, particularly given recent findings showing disparities in labor pain management among racial and ethnic minorities.^39^, ^44^ Recent findings show that Latinas report poor labor pain management, as measured by fewer administered medications.^45^ The disparity in pain management among Latinas is significant because this group may also practice cultural beliefs about pain management that reduce their likelihood of taking pain medication and medication adherence. For example, the concept of Marianismo suggests that the woman must endure suffering for her children and family, including during childbirth.^14^ Studies also show that Latinas believe pain is a critical aspect of childbirth.^46^ Others have found that Latinos report negative perceptions of pain medications.^47^ Thus, clinicians must offer an alternative to pharmacologic pain management that aligns with Latinas’ preferences while enabling them to feel in control of childbirth labor.

In summary, our novel study identified 3 critical factors associated with perceived control of childbirth labor. We found prenatal depressive symptoms, a history of pregnancy loss, and intrapartum pain severity were significantly and inversely associated with perceived control of labor in our sample of Latinas with vaginal childbirths. These findings underscore the critical role that patient histories and intrapartum experiences play in our participants’ childbirth experiences. The findings point to the need for further investigation to uncover the mechanisms by which these factors affect childbirth experiences and to identify potential intervention points.

### Limitations

To our knowledge, this is the first study to examine the association between pregnancy loss, prenatal depressive perinatal factors, and perceived labor control among Latinas. Still, it has limitations. First, the labor‐related data were based on retrospective participant reports, which may be subject to bias. Therefore, investigators are encouraged to collect labor and childbirth data immediately after childbirth. Second, the study did not include information from medical records, such as details on labor analgesia, including the use of nitrous oxide. Future studies should incorporate these data to minimize potential participant bias, recall bias, and inaccurate information. Similarly, investigators should collect pain‐related data and pain coping strategies (eg, use of a doula) during and immediately after childbirth, when health care professionals typically assess pain during labor, to reduce potential recall bias and fully capture pain‐reducing strategies. Third, other labor‐related data, such as beliefs about labor pain and pain catastrophizing, were not collected. This is critical, given that prior research shows that Latinas may have specific beliefs about pain during childbirth.^46^ Fourth, our study did not include data from hospitals where women gave birth or hospital practices related to factors, such as analgesia, that can influence the birth experience. Future studies should consider hospital‐level factors. Fifth, the data were collected between 2008 and 2012, making it challenging to contextualize current childbirth experiences, especially given the changes brought about by the coronavirus pandemic and current federal policies. Researchers should examine whether health care providers now offer better pain management approaches in the time since these data were collected and how those changes might affect Latina women's perceived control of labor today. Relatedly, data on country of origin, immigrant generation, and language preference were unavailable, limiting our ability to evaluate the potential role of these variables in perceived control over labor. This is critical, given observed disparities in perinatal pain management among racial and ethnic minorities, including Latinas.^39^, ^44^ Sixth, this study used a single item to measure perceived control of labor. Subsequent studies should use more comprehensive measures to fully capture a sense of control during childbirth, such as Perceived Labor Agentry, which assesses various aspects of perceived control.^13^ Incorporating the suggested variables will enhance the explained variance in future models; this study accounted for 28% of the variability in the dependent variable. Lastly, the results should be interpreted with caution because they are not generalizable to all Latinas with vaginal births, including those outside the geographic areas included in this study and other populations. Despite these limitations, this investigation underscores the importance of assessing and addressing pregnancy histories, mental health, and perinatal factors to improve Latinas’ intrapartum experiences. The findings offer opportunities to address critical gaps in care and inspire proactive research questions that offer promising health outcomes for Latinas. Future investigations should consider language and cultural differences among Latinas, given their varied experiences.

### Clinical and Health Care Policy Implications

The findings highlight key psychological and perinatal factors that shape the experiences of Latinas during vaginal births, providing concrete opportunities for clinical intervention to improve intrapartum care. For example, given the robust association between pregnancy loss and lower perceived control of childbirth labor, clinicians are encouraged to discuss their patients’ pregnancy histories to better anticipate intrapartum experiences and to offer the necessary resources and support that can enhance their sense of control during vaginal births. The results of this study also highlight the crucial need to identify depression symptoms and provide treatment before childbirth, creating opportunities for clinicians and researchers to reduce potential adverse effects during labor. Therefore, clinicians must screen for these symptoms and refer patients to mental health care professionals who can provide appropriate treatment options before childbirth. Health care policies must provide referrals to accessible mental health care services to ensure patients receive the prenatal behavioral care needed. Because pain severity can significantly reduce perceived control of childbirth labor, clinicians are advised to discuss pain management strategies during labor to help improve their patients’ sense of control during childbirth. Health care systems must provide patients with a variety of evidence‐based intrapartum pain management strategies and ensure that clinicians are responsive to adequately address pain.

## CONFLICT OF INTEREST

The authors declare no conflicts of interest.

## Supporting information




**Appendix S1**. Strengthening the Reporting of Observational Studies in Epidemiology (STROBE) Checklist
